# MetaPhinder—Identifying Bacteriophage Sequences in Metagenomic Data Sets

**DOI:** 10.1371/journal.pone.0163111

**Published:** 2016-09-29

**Authors:** Vanessa Isabell Jurtz, Julia Villarroel, Ole Lund, Mette Voldby Larsen, Morten Nielsen

**Affiliations:** Department of Systems Biology, Technical University of Denmark, Lyngby, Denmark; Iowa State University College of Veterinary Medicine, UNITED STATES

## Abstract

Bacteriophages are the most abundant biological entity on the planet, but at the same time do not account for much of the genetic material isolated from most environments due to their small genome sizes. They also show great genetic diversity and mosaic genomes making it challenging to analyze and understand them. Here we present MetaPhinder, a method to identify assembled genomic fragments (i.e.contigs) of phage origin in metagenomic data sets. The method is based on a comparison to a database of whole genome bacteriophage sequences, integrating hits to multiple genomes to accomodate for the mosaic genome structure of many bacteriophages. The method is demonstrated to out-perform both BLAST methods based on single hits and methods based on k-mer comparisons. MetaPhinder is available as a web service at the Center for Genomic Epidemiology https://cge.cbs.dtu.dk/services/MetaPhinder/, while the source code can be downloaded from https://bitbucket.org/genomicepidemiology/metaphinder or https://github.com/vanessajurtz/MetaPhinder.

## Introduction

Bacteriophages, phages in short, are viruses that prey on bacteria. With an estimated total number of 10^31^ particles [1], they constitute the most abundant biological entity on earth. Even though the phages *MS*2 and *ΦX*174 were the first organisms ever to be sequenced in full [2] [3], this did not spur the scientific interest in phages at the time, beyond their use for deducing several central principles within molecular biology [4]. This is currently changing, as an increasing amount of problems with antibiotic resistant bacterial strains are encountered [5]. Phages, as the natural enemies of bacteria, are highly interesting candidates to replace antibiotics in many settings. Phage therapy has been applied as a successful treatment in the states of the former Soviet Union for decades and is now being increasingly researched around the world [6–9]. Apart from their therapeutic potential, phages have huge impacts on the environment as catalysts for biogeochemical cycling, affecting the nutrient cycling in the ocean by killing a large part of the bacterial population every day [1]. While phages outnumber their bacterial hosts approximately 10:1 they only contribute 2-5% of the DNA found in most environments, due to their small genome size [10]. At the same time they are the genetically most diverse organisms on the planet and we are only beginning to understand their genome space [1]. There is no single gene that is shared by all phages and it is therefore often hard to draw the line between phages and mobile genetic elements [11]. Before genomic sequencing became widely available, phages were assigned taxonomic labels based on their nucleic acid type (dsDNA, ssDNA, dsRNA or ssRNA) and the virion morphology [12]. Taxonomic phage families differ in genome size due to space limitations in the virion. Furthermore, some phages have a highly mosaic genome structure, making their taxonomic classification difficult [13]. All of these factors complicate the study of phages in metagenomic samples.

Different approaches have been adopted in studies focusing on the viral fraction of metagenomic samples. An indirect approach to studying phages, is to extract CRISPR sequences from bacterial genomes. CRISPR sequences are part of a bacterial defense system against phages and can give insight to previous encounters between phages and bacteria [14] [15].

In some studies, the phage particles are physically separated from the larger cells by an extensive series of filtration and ultracentrifugation steps before sequencing [16–18]. It is, however, often difficult to obtain a pure viral fraction devoid of any genetic material from cells. Further, current protocols for extracting the viral particles lead to different yields as well as bias towards certain types of phages [19].

Another approach is to sequence the genetic material of the entire metagenomic sample without any prior separation. The resulting sequence reads can be assembled into contigs, which must then be assigned to taxonomic groups. This can be done by comparison to several databases like the ACLAME database [20], the Antibiotic Resistance Genes Database ARDB [21], Virulence Factors Database VFDB [22], and the Phage Orthologous Groups POG [23]. Contigs are then identified as of phage origin by the presence of a number of well-known phage genes. The following studies are examples that follow this approach: [24] [25] [26] [27] [28]. Previous methods for the automatic characterization of metagenomic fragments include PhyloPythiaS [29], MEGAN [30] and MG-RAST [31]. None of them have, however, been optimized for the identification of fragments of phage origin. Other tools have been developed for the analysis of the assembled virome, but assumes that the input data is all of virus origin [32]. A number of tools have been developed to annotate prophage sequences in bacterial genomes, including PhiSpy [33] and phage finder [34]. PhiSpy relies on several criteria to identify prophages including protein length, transcription strand directionality, AT and GC skew, presence of phage insertion sequences and similarity to phage proteins. These criteria are evaluated by a random forest to make the final prediction of prophages. Phage finder identifies prophage regions based on a comparison to phage-specific hidden markov models (built using phage proteins).

An alternative approach that does not require comparison to a database to separate metagenomic data into viral and bacteria species has been suggested by Nielsen et al [35]. This method is based on a comparison of the abundance of genes over several metagenomic samples and the identification of co-abundant gene groups CAGs. This approach has the large advantage of being independent of a database, especially in cases where a large part of the genomic diversity remains unsequenced. However multiple similar metagenomic samples that allow the determination of CAGs are not available in every study, so we believe there is a need for methods that can be applied to single sequencing experiments, like the here presented method. Further, it is still necessary to determine if a CAG is of phage origin or not, which involves database comparisons.

Here we present a method to extract phage contigs from previously (*de novo*) assembled metagenomic contigs. The aim is to provide an easy to use and fast approach for selecting potentially interesting contigs relying on a tested criterion. We establish a database of known phage whole genome sequences and search it using blastn. The blastn results are then combined to account for the mosaic genome structure of phages and a criterion is applied to classify a contig as of phage origin or non-phage. The method is available as a webservice here: https://cge.cbs.dtu.dk/services/MetaPhinder/ and the code can be downloaded here: https://bitbucket.org/genomicepidemiology/metaphinder.

## Methods

### Data set preparation

#### Phage data set

A data set of phage whole genome sequences (WGS) was downloaded in August 2014 from publicly available sources. A unique list of IDs for upload to the Batch Entrez service of NCBI was obtained from Phages.ids—VBI mirrors page (http://mirrors.vbi.vt.edu/mirrors/ftp.ncbi.nih.gov/genomes/), the NCBI viral Genome Resource (http://www.ncbi.nlm.nih.gov/genomes/GenomesHome.cgi), the EMBL EBI phage genomes list (http://www.ebi.ac.uk/genomes/phage.html), the phagesdb databases for Mycobacteriophages (http://phagesdb.org/), Arthrobacter (http://arthrobacter.phagesdb.org/), and Bacillus (http://bacillus.phagesdb.org/), and Streptomyces (http://streptomyces.phagesdb.org/). Additionally, WGS were downloaded from the PhAnToMe genomes database (http://www.phantome.org) and from NCBI searching for’(phage[Title]) AND complete genome’. After manual curation, 2495 phage WGS remained in the data set. In a next step homology reduction was applied to eliminate redundancies and bias. Genomes with over 90% average nucleotide identity (ANI) to a genome already included in the database were excluded using a hobohm 1 algorithm [36]. %ANI was determined as shown in Eq (1) based on blastn [37] results. In Eq (1) *N* is the number of blastn hits between one query sequence and one subject sequence in the database, *id* is the % identity value reported by blastn, *al* is the alignment length reported by blastn, and *m*_*cov*_ is the coverage of the query sequence by all hits after overlapping hit regions have been merged.

$$\%ANI=\frac{\sum_{i=1}^{N}id_{i}\cdot al_{i}}{\sum_{i=1}^{N}al_{i}}\cdot m_{cov}$$ (1)

Homology reduction resulted in a data set of 1534 phage whole genome sequences.

Subsequently, the data set was divided into five partitions by sorting the genomes according to genome size and randomly assigning five subsequent genomes to each of the five partitions. Sorting by genome size was applied to obtain a comparable distribution of taxonomies over the different data partitions.

#### Negative data set

A negative data set of bacterial whole genome sequences (bacterial chromosomes and plasmids) was downloaded from the NCBI ftp page (ftp.ncbi.nlm.nih.gov/genomes). Homology reduction was performed using KmerFinder [38] [39], which also relies on a hobohm 1 algorithm [36]. Blastn was not applied here due to the extremely long runtime required for analyzing this data set of bacterial sequences. KmerFinder extracts all possible sequences of length k from a query DNA sequence and determines the similarity of the query sequence to all sequences in a database by counting the number of identical k-mers. KmerFinder’s k-mer size was set to 16, the prefix to ATG to reduce the total amount of k-mers. The homology threshold was set to 0.44 to ensure that all sequences with an ANI of 95% or more were excluded, according to equation Eq (2). Where *q*_*cov*_ is the amount of identical k-mers in query and template sequence divided by the amount of unique kmers in the database and *k* is the k-mer size.

$$q_{cov}={\left(\frac{\%ANI}{100}\right)}^{k}$$ (2)

Eq (2) assumes that mutations are randomly distributed across the genome. Another possibility is that mutations cluster in a specific region (or a region is inserted/deleted). In the case of clustering/consecutive mutations *q*_*cov*_ should be identical to the %*ANI*. We built a phage database using a hobohm1 algorithm that adds phage genomes iteratively to the database, and reports the KmerFinder *q*_*cov*_ to the most similar phage in the database each time a new phage is added. Subsequently we calculated the corresponding blastn %ANI between each of these phage pairs and plotted the similarities found by Kmer-Finder against the %ANI obtained with blastn in S1 Fig. In this way it was verified that Eq (2) can be used to determine the maximal possible %ANI value given a specific *q*_*cov*_ reported by Kmer-Finder.

Also here the bacterial genetic entities (chromosomes or plasmids) were sorted by genome size before partitioning to obtain a comparable number of plasmids in all data partitions. As additional negative data sets, fungi, protozoa, other viruses and a human genome were downloaded from the ncbi ftp site (see above). Random pieces of these sequences (corresponding to the phage genomes in length) were cut out and used as negative examples. Due to the limited amount of completely sequenced genomes and the process of cutting out pieces of the sequences, it was not necessary to perform homology reduction. In this way, for each of the five phage data partitions a negative counterpart was made with the same amount of sequences (50% bacterial and 50% other negative examples).

#### Artificial contigs data set

To further mimic metagenomic contigs, pieces of random length (min. 500 bp) were cut out of each phage genome five times. The same procedure was applied to the negative data set, requiring the cuts to be of the same size as the phage cuts. This was performed after partitioning of the original data and the artificial contigs were assigned to the partition they were derived from.

### Method development

A BLAST database was built from partitions 1-4 of the phage data set (negative data and artificial contigs were not included in the database). Subsequently this database was searched using blastn [37] with data partitions 5 of the phage, negative and artificial contigs data sets as queries. This process was repeated five times such that each phage partition was used as query to search a phage-BLAST-database once. The %ANI of a query sequence to the whole database was calculated, based on all hits with an e-value of 0.05 or smaller, and used to classify phage/non-phage sequences. The similarity to the whole database rather than the similarity to the best hit was used to account for the mosaic genome structure of some phages. This approach was compared to classifying query sequences as phage/non-phage based solely on the e-value of the top hit only for each query sequence. Additionally, the phage database was searched using tBLASTx [40] (applying an e-value of 0,05)), which searches a translated nucleotide database using a translated nucleotide query, and KmerFinder with a k-mer size of 16 and a prefix of AT and then determining the query coverage to the whole database.

The predictions on the five data partitions were combined to assess performance, instead of calculating performance values separately on each partition and reporting a mean performance value. Subsequently AUC (area under the ROC curve) was calculated in R using ROCR [41] and visualized using ggplot2 [42]. To further investigate the performance, the data set of positive and negative examples was split into subsets depending on sequence length and the AUC was calculated separately for each of these partitions. Finally a classification threshold was found by setting the false positive rate to be equal to 1-true positive rate.

### Method verification

The final method was evaluated on different publicly available data sets:

A data set of manually curated prophages was downloaded from the PhanToMe website [43]. Further a data set of conserved prophages predicted using PhiSpy [33], which was created by Kleinheinz et al. [44] was downloaded and analyzed.

The data set of metagenomic co-abundance gene groups (CAGs) published by Nielsen et al. [35] was downloaded and MetaPhinder predictions were compared to the annotations of phagelike CAGs made by Nielsen et al.

## Results

### Data set partitioning

**Fig 1:**
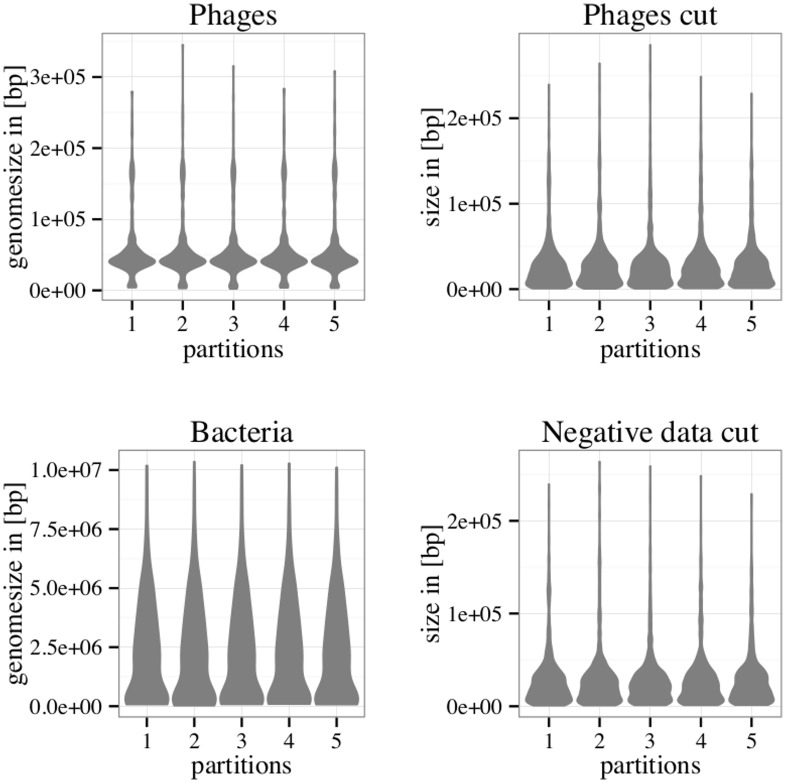
Size distribution of the phage and bacterial genomes and the cuts made to imitate metagenomic contigs.

The phage data set, negative data set and the artificial contigs data set were each partitioned into five subsets as described in Methods. As seen in Fig 1, all five partitions of the phage data are similar in terms of sequence length. The same is true for the partitions of the negative data set. Further, all partitions of the artificial contigs data sets (phage and negative) are comparable to each other in terms of sequence length.

### MetaPhinder classification threshold selection and performance

**Fig 2:**
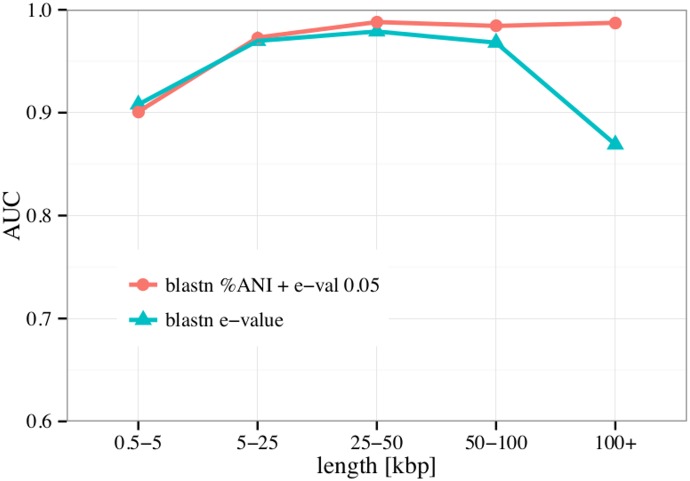
Performance according to sequence length. The performances measured in AUC for %ANI classification and top-hit e-value classification are compared. Data are binned according to sequence length and performance is shown separately for each bin. Note that the amount of sequences in each bin differs but the amount of positive and negative examples is always comparable.

Initially, the performance of a method that classified sequences as phage/non-phage based on %ANI was compared to a method based on the e-value of the top hit found by blastn. For this purpose, the complete predictions data set (phages, negative data and artificial contigs) was partitioned according to sequence size and AUC values were calculated separately for different size ranges as shown in Fig 2. From this analysis, it was clear that the %ANI to the whole database outperformed the top 1 hit approach for longer sequences and that the two approaches performed comparable for shorter sequences. Overall the performance was higher for longer sequences and droped markedly for query sequences shorter than 5000 base pairs. S2 Fig shows that applying different e-value cutoffs for accepting hits to contribute to the %ANI calculation does not lead to large variations in performance. Only does a too stringent e-value lead to a decreased performance when classifying shorter sequences. S3 Fig compares the %ANI obtained when looking only at the top BLAST hit with the %ANI obtained by including all hits with an e-value equal to or less than 0.05. Taking into account all hits to the phage database is advantageous when dealing with mosaic phages. An example of such a phage is given in S4 Fig where the coverage of the phage NC 018085 (Bacillus phage BtCS33) by the top five most similar phages in the database is visualized.

**Fig 3:**
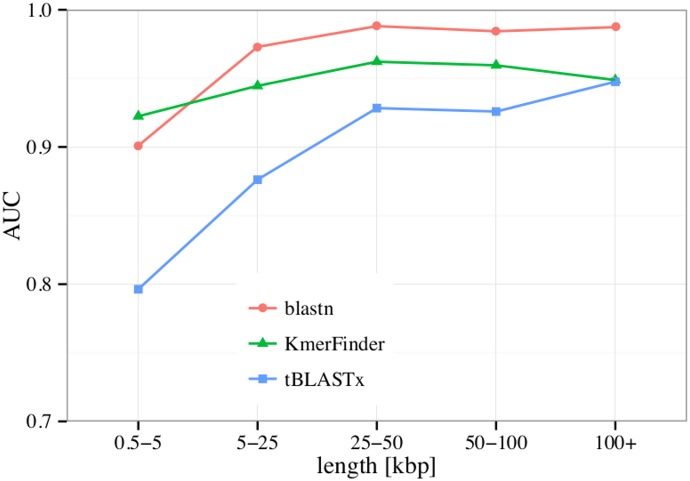
Comparison of different methods used to search the whole genome phage sequences database. For BLAST and tBLASTx %ANI is calculated to determine similarity to the database, whereas for KmerFinder the query coverage (*q*_*cov*_) is applied. Performance is evaluated based on AUC. Data are binned according to sequence length and performance is shown separately for each bin.

The approach of using BLAST to search the phage whole genome database was further compared to using tBLASTx or KmerFinder and results are shown in Fig 3. This comparison shows that the BLAST approach clearly outperforms tBLASTx for all contig sizes. For small contigs of less than 5000 base pairs KmerFinder has a slight advantage over BLAST. For larger contigs KmerFinders performance remains below that of BLAST.

**Fig 4:**
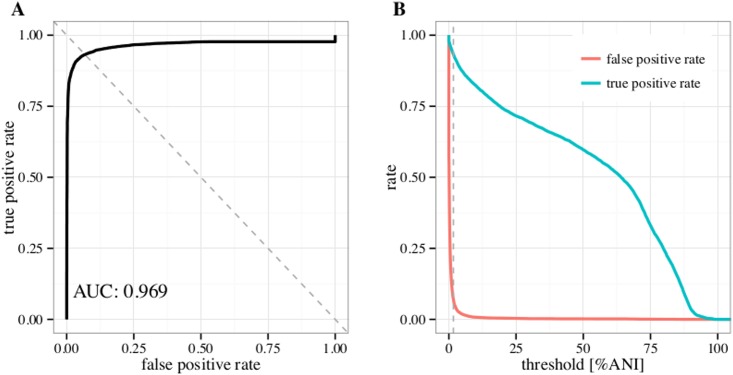
MetaPhinder performance curves. (A) ROC curve intersected by the dashed line used to select the classificaction threshold. (B) True positive rate and false positive rate compared for different classification thresholds (in %ANI to the whole phage database). The vertical dashed line indicates the selected classification threshold.

Taking the above results into account it was decided to use BLAST to search the phage whole genome database using an e-value threshold of 0.05. A classification threshold in %ANI for the final method was selected by combining the predictions on all partitions of the phage, negative and artificial contigs data sets and requiring the false positive rate to be equal to 1-true positive rate. This corresponds to intersecting the ROC curve with the dashed line shown in Fig 4(A) and resulted in a classification threshold of 1.7%ANI. This threshold was found to be stable when calculated on the five data partitions separately (1.762 ± 0.220 %ANI) or on data partitions with different sequence length ranges (1.733 ± 0.157 %ANI). Fig 4(B) gives an overview of the true positive rate and false positive rate for different classification thresholds. A more detailed summary of MetaPhinders predictions on different datasets (i.e. phages, bacteria, other negative data) values are given in Table 1. While the number of hits is high in the bacteria data, the merged coverage is very low, making the distinction of phages and bacteria possible. The high mean and median %ANI observed in the phage datasets suggests that the currently available phage genomes contain many entries with high mutual sequence similarities.

**Table 1:** MetaPhinder performance measures and predictions for different data subsets. The data sets are described in detail in the the methods section, for phages we show performance on the whole genome data and artificial contigs data sets, for negative data we show the entire negative data, only bacteria sequences and negative data excluding bacteria (original and artificial contigs data sets for all three). Note, that the number of hits refers to the amount of database phages which were matched to the query sequence (not the total number of blastn alignments). For the positive phage and phage art. contigs data sets, the sensitivity was calculated and for the other negative data sets the specificity.

data	mean number of hits	median number of hits	mean %ANI	median %ANI	mean merged coverage	median merged coverage	sensitivity	specificity
phages	68.554	61	51.918	63.543	0.645	0.819	0.948	-
negative all	68.019	2	0.495	0.111	0.006	0.001	-	0.963
negative bacteria	135.327	157	0.937	0.355	0.012	0.005	-	0.927
negative excl. bacteria	0.447	0	0.079	0	0.001	0	-	0.996
phages art. contigs	45.292	32	52.276	64.246	0.649	0.833	0.930	-
negative all art. contigs	4.116	1	0.723	0.097	0.009	0.001	-	0.922
negative bacteria art. contigs	6.937	2	1.202	0.269	0.015	0.003	-	0.873
negative excl. bacteria art. contigs	1.310	0	0.244	0	0.003	0	-	0.971

For the final version of MetaPhinder a database was created containing the entire homology reduced phage whole genome data set. This database is then searched using blastn with an e-value threshold of 0.05 and the %ANI of each query contig to the whole database is calculated according to Eq (1). The %ANI threshold to classify a contig as of phage origin is set to 1.7%ANI.

### Method verification

To further evaluate the accuracy of the method, predictions on different prophage data sets were made using MetaPhinder. Two data sets of manually curated prophages were downloaded from the PhanToMe website [43]. Prophage predictions were initially made on a dataset of bacterial genomes with PhiSpy [33] and phage finder [34] and subsequently manually curated. Of the 139 manually curated prophages based on predictions by PhiSpy 134 were correctly identified as phages, and in the case of the manually curated prophages based on predictions by phage finder 120 out of 122 were predicted correctly. Additionally, a larger data set of conserved PhiSpy predictions (not manually curated) published by Kleinheinz et al. [44] was analyzed. Here 1124 out of 1442 prophages were predicted correctly by MetaPhinder. An overview of the predictions is given in Table 2.

**Table 2:** Results of MetaPhinder predictions on different prophage data sets.

Data set	predicted as phage	total amount
manually curated prophages PhiSpy	134 (96.4%)	139
manually curated prophages phage finder	120 (98.4%)	122
conserved prophages PhiSpy	1124 (77.9%)	1442

Further the data set of metagenomic co-abundance gene groups (CAGs) published by Nielsen et al. [35] was analyzed with MetaPhinder. The data set contains 741 metagenomic species (MGS), which are defined as larger CAGs of more than 700 genes related to bacterial species, as well as 6640 smaller CAGs. None of the MGS were predicted to be of phage origin by MetaPhinder. Of the 6640 smaller CAGs, 884 were predicted to be of phage origin by MetaPhinder, whereas Nielsen et al. for these CAGs annotate 848 as phagelike. Both methods (MetaPhinder and the one applied by Nielsen et al.) agree on 387 CAGs to be phagelike. The 80% percentile score of MetaPhinder on these 387 CAGs is 8.18%ANI whereas the 80% percentile score of CAGs predicted as of phage origin only by MetaPhinder is 5.11%ANI. Moreover is the maximum %ANI for these latter CAGs 88.7%ANI. In contrast to this is the 80% percentile score for the CAGs labeled as phagelike only by Nielsen et al. as low as 1.18%ANI, and the maximal score is 1.7%ANI. These numbers strongly suggest that at least part of the CAGs missed by the method by Nielsen et al. are indeed phagelike and that a large proportion of the phagelike CAGs predicted by Nielsen et al. are either novel phages with very limited similarity to known phages or false positive predictions.

## Discussion

Here we present a method to identify contigs of phage origin in metagenomic data. The method is available for download as well as an online service at the Center for Genomic Epidemiology https://cge.cbs.dtu.dk/services/MetaPhinder/.

MetaPhinder identifies contigs of phage origin by comparing a query contig to a database of phage whole genome sequences. Blastn was selected to search the database, since it outperformed tBLASTx and KmerFinder. The MetaPhinder method was successfully applied to data sets of prophage genomes and the co-abundance gene groups derived from a metagenomic data set of human gut microbiome samples.

The MetaPhinder method is based on a comparison of contigs to a database of whole genome DNA sequences of phages. All hits are combined into an average nucleotide identity (%ANI) which can be an advantage in the case of mosaic genomes and genome rearrangement. Further it enables one to distinguish bacteria with prophages from phages (as seen when comparing to the top hit e-value approach). It can be argued that one should rather compare amino acid sequences of proteins, since remote similarities can still be detected in the amino acid sequence that are hard to determine in the underlying DNA sequence. Further Kristensen et al. [23] demonstrated that many of the phage orthologous groups POGs are specific to phages and never found in bacterial genomes outside of prophage regions. Kristensen et al. reported that around 50-70% of all proteins in a viral genome are represented in POGs. Very remote similarities to a single POG might indicate a contig of phage origin, but it is hard to distinguish phage sequences from bacterial sequences containing a prophage using this criterion. We hypothesize that this is the reason why we do not see an increase in performance when using tBLASTx instead of blastn to search the phage database. We also tested KmerFinder as an alternative search algorithm but found blastn to result in better classification performance for contig sizes above 5000 base pairs. KmerFinder is unable to detect similarities in stretches of DNA where mutations occur closer together than the selected k-mer size, which might be disadvantageous for identifying contigs of phage origin.

The classification threshold we find, requiring 1.7%ANI to the phage database to classify a contig as of phage origin, is very low due to the large amount of presently unknown phage genes. It is likely that the classification threshold will shift as more phage genomes become available.

Apart from the performance we obtained in our cross-validation setup we tested the method on prophage data sets. If prophages are taken out of the context of the bacterial genome they are integrated into, a phage sequence should remain and be classified as such by MetaPhinder. The results on the manually curated data sets show that more than 95% of the prophages can be identified as phages. This result does not necessarily indicate that there is large agreement between MetaPhinder and PhiSpy or phage finder, since the prophage genomes were manually curated after prediction with PhiSpy or phage finder. Of the prophages predicted by PhiSpy without manual curation (published by Kleineheinz et al. [44]) only 77.9% can be identified as phage sequences by MetaPhinder. PhySpy classifies prophage sequences based on several criteria of which some are not related to database comparisons, i.e. gene length and transcription strand directionality, and can therefore also identify novel prophages without sequence similarity to previously sequenced phages. However, it is hard to argue the correctness of the prediction of novel phages by PhiSpy, and PhiSpy predictions have been observed to vary greatly between different runs [44], therefore it is conceivable that some of its predictions are false.

When predicting CAGs both MetaPhinder and the method proposed by Nielsen et al [35] predict a similar number to be of phage origin (884 by MetaPhinder and 848 by Nielsen et al.). However, the two methods agree on less than 50% of these predictions. Many possible reasons for this low concordance exist. The merged CAGs are not real contigs and therefore might be more difficult for MetaPhinder to predict. Additionally, Nielsen et al. used a criterion based on comparing CAG protein sequences to known phage proteins, which might pick up on remote similarities that cannot be utilized to distinguish phages from bacterial sequences. MetaPhinder is not able to identify novel phages, it requires a minimal similarity of 1.7%ANI to known phages to identify contigs of phage origin. However, of the additional 497 CAGs that MetaPhinder predicts as of phage origin, many have very high %ANI to the phage database, indicating that MetaPhinder is able to identify phage sequences that would be missed if one only compares to POGs, since they only cover part of the known phage genosphere. Many of the CAG predicted only by the Nielsen method share extremely low similarity to the database of known phage genomes suggesting that some of these predictions could be of low reliability.

Overall we have successfully developed a method that is able to identify metagenomic contigs of phage origin based on similarity to a database of phage whole genome sequences. The method performs very well with an AUC of 0.969. The method was further tested on prophage data sets, where it also performed well. While a sufficient degree of similarity to the phage database is required to be able to identify phage contigs, the here presented method makes use of as much of the known phage diversity as possible by using whole genome sequences as opposed to conserved protein families.

## Supporting Information

S1 Fig Comparison of KmerFinders query coverage *q*_*cov*_ and %ANI calculated based on blastn results. Phages were iteratively added to a database and the *q*_*cov*_ and %ANI to the most similar phage in the database are plotted.
(TIF)

S2 Fig Comparison of different e-value thresholds for accepting hits to contribute to the calculation of the %ANI to the whole phage database. (TIF)

S3 Fig Comparison of the %ANI to the top blastn hit and the whole phage database. (TIF)

S4 Fig Visualization of the comparison of phage NC 018085 (Bacillus phage BtCS336) to the phage database. Only the top five most similar phage genomes in the database are shown, in total hits to 35 phage genomes were found.
(TIF)
