# Supplementary figures and images for: MetaPhinder—Identifying Bacteriophage Sequences in Metagenomic Data Sets
Source: PLoS One. 2016 Sep 29;11(9):e0163111. doi: 10.1371/journal.pone.0163111 (PMC5042410; doi:10.1371/journal.pone.0163111)

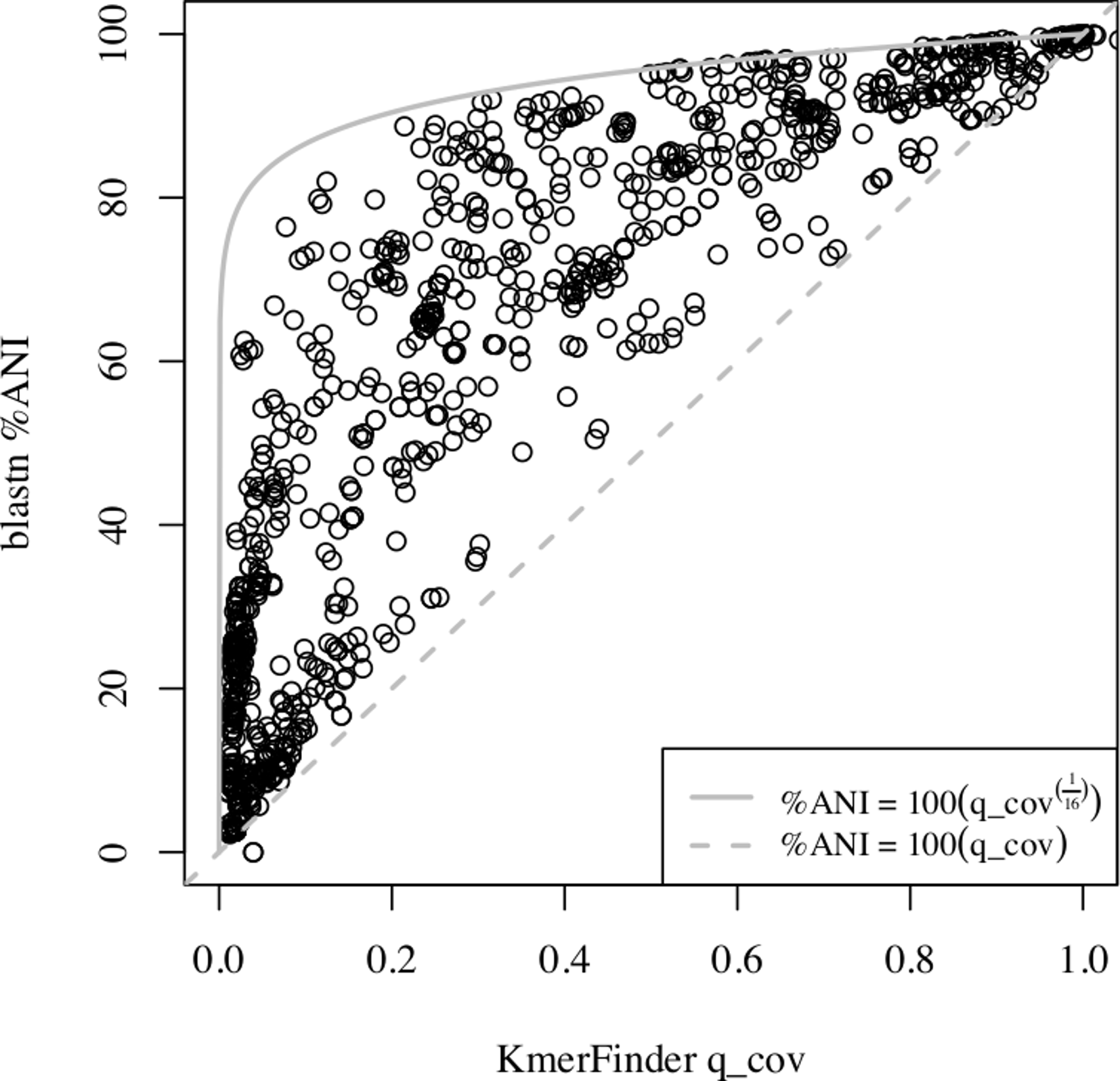

Supplement: S1 Fig — Phages were iteratively added to a database and the qcov and %ANI to the most similar phage in the database are plotted. (TIF) [file pone.0163111.s001.tif]

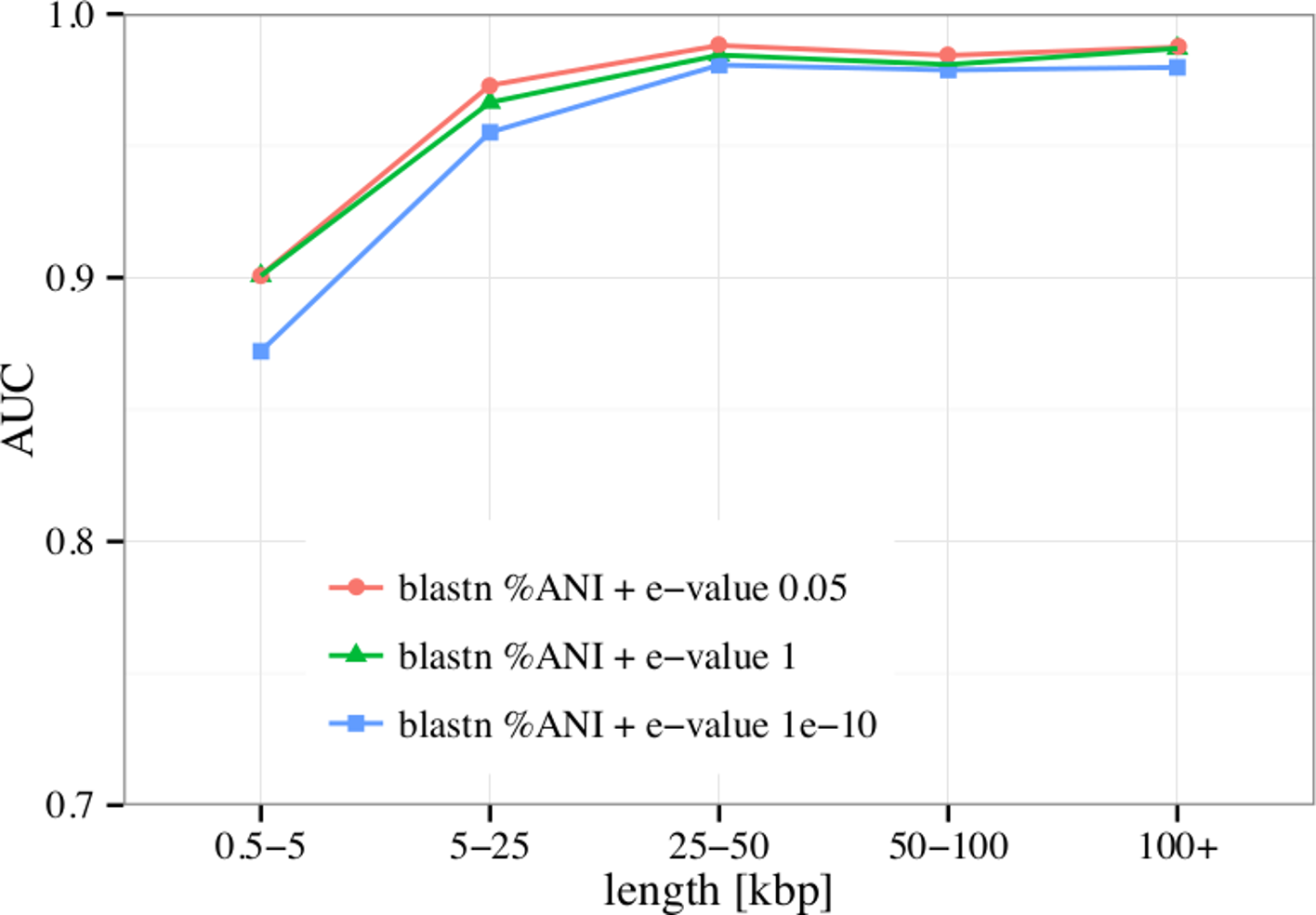

Supplement: S2 Fig — (TIF) [file pone.0163111.s002.tif]

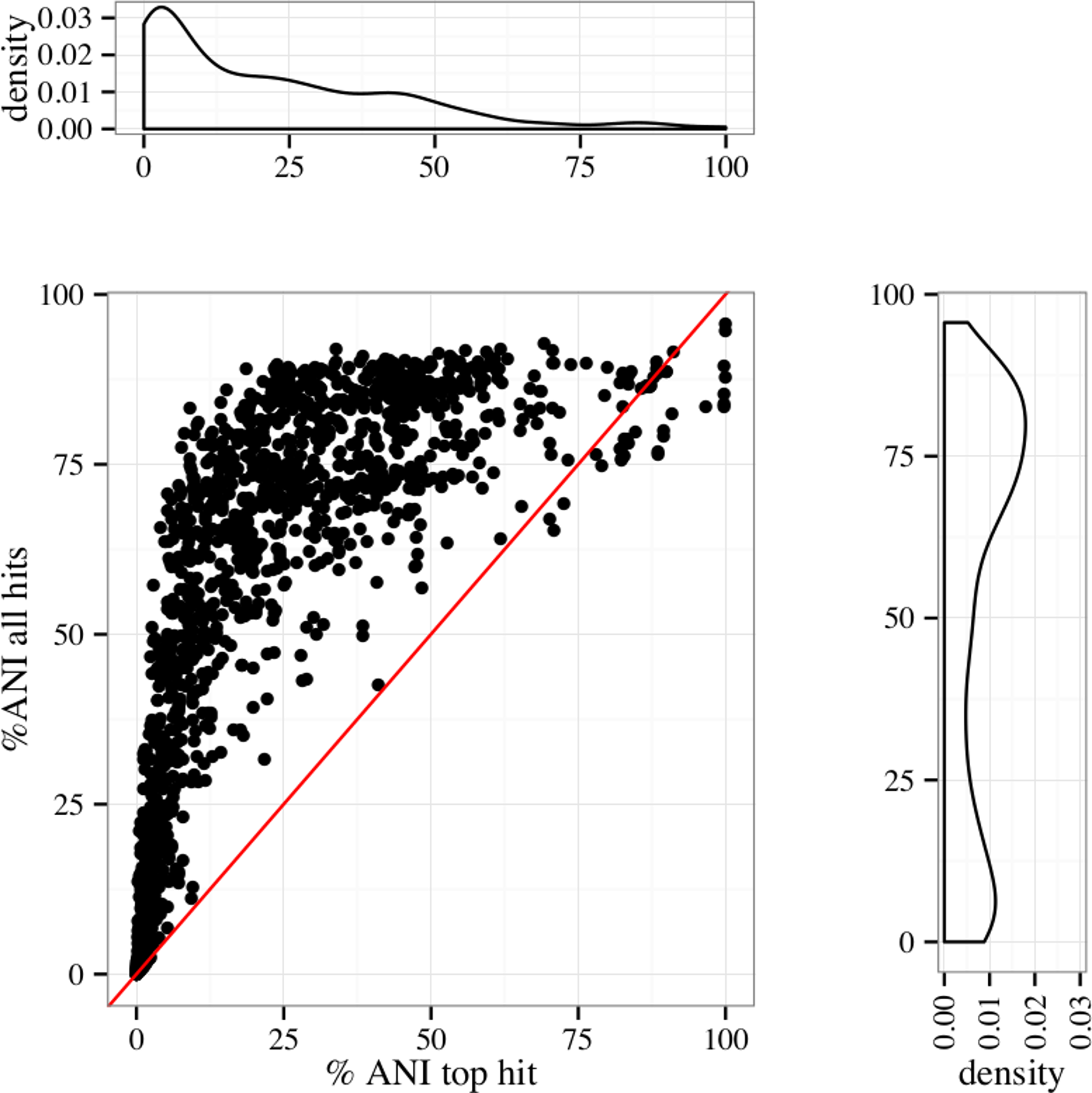

Supplement: S3 Fig — (TIF) [file pone.0163111.s003.tif]

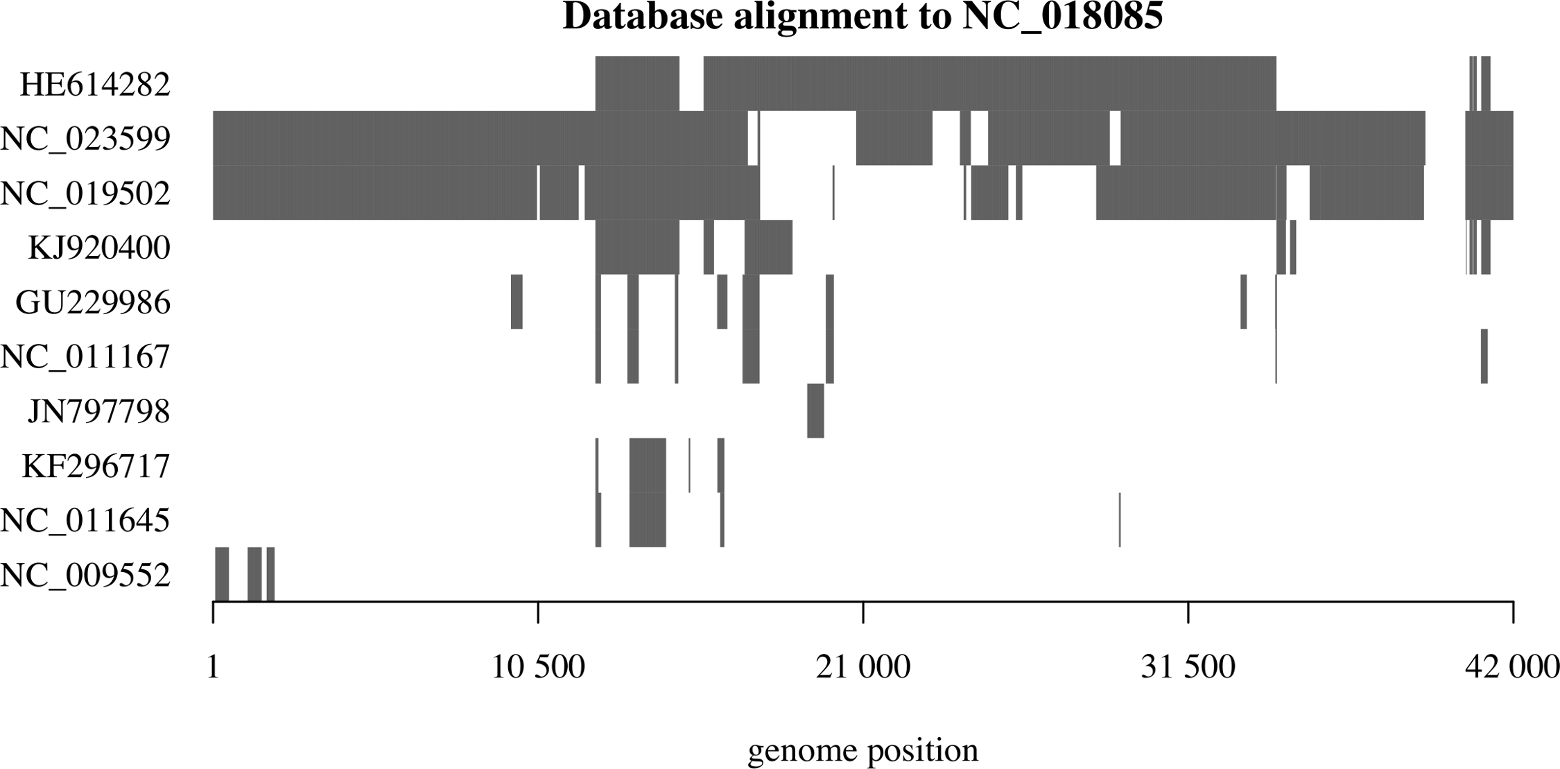

Supplement: S4 Fig — Only the top five most similar phage genomes in the database are shown, in total hits to 35 phage genomes were found. (TIF) [file pone.0163111.s004.tif]
